# Evaluation of feasibility, added value and satisfaction of remote digital self‐assessment for mild cognitive impairment in primary and specialized routine care with the app neotivCare

**DOI:** 10.1002/alz.088681

**Published:** 2025-01-03

**Authors:** Emrah Düzel, Michael Schoettler, Harriet Sommer, Martin Griebe

**Affiliations:** ^1^ German Center for Neurodegenerative Diseases (DZNE), Magdeburg Germany; ^2^ Institute of Cognitive Neurology and Dementia Research (IKND), Otto‐von‐Guericke University, Magdeburg Germany; ^3^ Roche Pharma AG, Grenzach‐Wyhlen, Baden‐Württemberg Germany; ^4^ Department of Neurology, University Medical Center Medical Faculty Mannheim, Heidelberg, Baden‐Württemberg Germany

## Abstract

**Background:**

Timely diagnosis of mild cognitive impairment (MCI) in Alzheimer’s disease (AD) is crucial for early interventions, but its implementation is often challenging due to the complexity and time burden of required cognitive assessments. Remote unsupervised self‐testing of cognition can potentially addres this health care challenge. We conducted the to date largest evaluation of feasibility and experienced added value of unsupervised digital remote assessment in primary and specialized health care in Germany.

**Method:**

This is a prospective, multicentric health care research evaluation survey called “re.cogni.ze”. Its goal is to evaluate physician satisfaction with a remote digital assessment solution (neotivCare) in primary and specialized routine care in Germany. Physicians in different regions of Germany have been recommending the app to approximately 1000 patients for a 12‐week self‐assessment of cognition. Last patient out is on 31st January 2024. The primary endpoint is the evaluation of physicians’ and patients’ overall satisfaction with neotivCare in comparison to neuropsychological questionnaires / standard procedures using a Likert scale. Secondary endpoints include user‐friendliness, qualitative assessment of acceptance, and potential improvements on medical routine services.

**Result:**

This is first large scale assessment of feasibility, satisfaction and experienced added value of remote digital self‐assessment of MCI in routine care. The data will be analysed according to prespecified endpoints after data closure on the 31st January 2024.

**Conclusion:**

The study results will provide insights into the feasibility, efficiency, and acceptance of new digital tools for MCI diagnosis in routine care. The re.cogni.ze survey will thus provide proof‐of‐concept information for the implementation of remote digital cognitive assessment apps for MCI into medical routine care.

